# *Kaempferia parviflora* Extract Exhibits Anti-cancer Activity against HeLa Cervical Cancer Cells

**DOI:** 10.3389/fphar.2017.00630

**Published:** 2017-09-11

**Authors:** Saranyapin Potikanond, Siriwoot Sookkhee, Mingkwan Na Takuathung, Pitchaya Mungkornasawakul, Nitwara Wikan, Duncan R. Smith, Wutigri Nimlamool

**Affiliations:** ^1^Department of Pharmacology, Faculty of Medicine, Chiang Mai University Chiang Mai, Thailand; ^2^Department of Microbiology, Faculty of Medicine, Chiang Mai University Chiang Mai, Thailand; ^3^Department of Chemistry, Faculty of Science, Chiang Mai University Chiang Mai, Thailand; ^4^Environmental Science Program, Faculty of Science, Chiang Mai University Chiang Mai, Thailand; ^5^Institute of Molecular Biosciences, Mahidol University Nakorn Pathom, Thailand

**Keywords:** anti-cancer, apoptosis, cervical cancer, invasion, *Kaempferia parviflora*, MAPK pathway, migration, PI3K/AKT pathway

## Abstract

*Kaempferia parviflora* (KP) has been traditionally used as a folk remedy to treat several diseases including cancer, and several studies have reported cytotoxic activities of extracts of KP against a number of different cancer cell lines. However, many aspects of the molecular mechanism of action of KP remain unclear. In particular, the ability of KP to regulate cancer cell growth and survival signaling is still largely unexplored. The current study aimed to investigate the effects of KP on cell viability, cell migration, cell invasion, cell apoptosis, and on signaling pathways related to growth and survival of cervical cancer cells, HeLa. We discovered that KP reduced HeLa cell viability in a concentration-dependent manner. The potent cytotoxicity of KP against HeLa cells was associated with a dose-dependent induction of apoptotic cell death as determined by flow cytometry and observation of nuclear fragmentation. Moreover, KP-induced cell apoptosis was likely to be mediated through the intrinsic apoptosis pathway since caspase 9 and caspase 7, but not BID, were shown to be activated after KP exposure. Based on the observation that KP induced apoptosis in HeLa cell, we further investigated the effects of KP at non-cytotoxic concentrations on suppressing signal transduction pathways relevant to cell growth and survival. We found that KP suppressed the MAPK and PI3K/AKT signaling pathways in cells activated with EGF, as observed by a significant decrease in phosphorylation of ERK1/2, Elk1, PI3K, and AKT. The data suggest that KP interferes with the growth and survival of HeLa cells. Consistent with the inhibitory effect on EGF-stimulated signaling, KP potently suppressed the migration of HeLa cells. Concomitantly, KP was demonstrated to markedly inhibit HeLa cell invasion. The ability of KP in suppressing the migration and invasion of HeLa cells was associated with the suppression of matrix metalloproteinase-2 production. These data strongly suggest that KP may slow tumor progression and metastasis in patients with cervical cancer. Taken together, the present report provides accumulated evidence revealing the potent anti-cancer activities of *Kaempferia parviflora* against cervical cancer HeLa cells, and suggests its potential use as an alternative way for cervical cancer prevention and therapy.

## Introduction

Cervical cancer is still one of the most common causes of all cancer deaths in women, especially in developing countries (McGuire, 2016). Human papillomaviruses (HPVs) can subvert cellular mechanism of growth control (Moody and Laimins, 2010) and activate the PI3K/AKT/mTOR signaling (Surviladze et al., 2013). Like most cancers, cervical cancer does not show any signs during early disease development (Canavan and Doshi, 2000). However, symptoms usually appear when the tumor causes vaginal discharge and bleeding, and other symptoms including pain or backache may occur in patients with metastasis (Petignat and Roy, 2007). Therefore, most patients who notice symptoms typically have later stages of tumor development that have frequently progressed too far for curative treatment.

Communication through signal transduction pathways is very important for regulating the balance between cell proliferation and cell death. The activation of cell growth and survival signal transduction pathways is stimulated via a growth factor binding to a specific growth factor receptor (Margolis, 1992). Epidermal growth factor receptor (EGFR) is an important regulator of cell proliferation (Olayioye et al., 2000) and upon growth receptor activation, diverse downstream pathways are further stimulated. Those crucial pathways include Ras/Raf/MEK/MAPK, phospholipase C, and STAT (Yarden and Sliwkowski, 2001). Moreover, stimulation of the EGF receptor causes strong activation of the phosphatidylinositol-3 kinase (PI3K)/AKT pathway for maintaining cell metabolism and enhancing cell survival and proliferation (Cantley, 2002; Vivanco and Sawyers, 2002). The commonest characteristics of most cancers are the amplification of growth and survival signaling, and the inhibition of apoptosis (Hanahan and Weinberg, 2011). Furthermore, elevated levels of matrix metalloproteinase (MMP) is considered to be an important hallmark of many cancers, and MMP expression has been demonstrated to be associated with tumor invasion in many different tumors (Liotta et al., 1980; Fishman et al., 1997; Stetler-Stevenson, 2001; Roomi et al., 2010). The actual cause of cancer is not well-understood. However, over activation and aberrant cancer cell signal transduction mediated by factors such as mutations in key kinases that cause constitutive activation of growth and survival signaling pathways, may contribute to cancer development and metastasis (Huang et al., 1997; Moscatello et al., 1998; Chakravarti et al., 2002).

The most effective way to decrease the burden of cervical cancer and the associated death rate is to prevent and screen for HPV lesions through HPV testing and Pap smears (Safaeian and Solomon, 2007). Vaccination is a better way to reduce and eventually prevent death from cervical cancer but this management option is currently limited to young people (Saslow et al., 2007). Moreover, in many underdeveloped countries routine screening may not be widely available due to limited resources (Denny et al., 2006). Although new chemotherapeutic agents have been developed to slow down the progression of the disease, the number of cancer related deaths is still high because of drug-resistance and metastasis (Liu et al., 2016; Siegel et al., 2016). Also, conventional treatments for cervical cancer are expensive (Subramanian et al., 2010). In particular, the direct cost per patient for cisplatin, placlitaxel, and topotecan treatments ranges from 2,000 to 10,000 USD (Geisler et al., 2012). Thus, many patients may not be able to afford these options.

Alternative medicine has emerged as an interesting means for treating or curing diseases, and recently several plants and their constituents have been approved to be safe, effective, and less expensive for managing the development and progression of various cancers (Yin et al., 2013). Several medicinal plants have been discovered to contain active compounds that are able to disrupt homeostasis of cancer cells (Yin et al., 2013). *Kaempferia parviflora* (KP) is a plant in the family Zingiberaceae commonly known as Thai black ginger (or Krachai Dam in Thai). Its rhizome is used in traditional medicine for many purposes including anti-gastric ulcer, anti-allergic, anti-plasmodial, and anti-cancer, as well as for enhancing sexual activity (Saokaew et al., 2016). Specifically, for the anti-cancer effects of KP, studies have shown that KP suppressed multidrug resistance associated proteins (MRP) in A549 (lung cancer) cells (Patanasethanont et al., 2007). Moreover, KP induced apoptotic cell death and enhanced paclitaxel or doxorubicin treatment in a promyelocytic leukemic cancer cell line (Banjerdpongchai et al., 2009). However, the anti-cancer effects of KP against cervical cancer cells have not yet been investigated. Several aspects, especially the molecular mechanisms of action to understand how KP interferes with growth and survival functions of cancer cells, remain largely unknown.

Thus, this present study aimed to evaluate the anti-cancer properties of KP against HeLa cervical cancer cells. We particularly investigated effects of KP on inducing apoptotic cell death, suppressing cell migration and invasion, and inhibiting major molecular signal transduction pathways related to cancer cell growth and survival. Our study provides convincing evidence that KP possesses anti-cancer properties and may be a good candidate as a new therapeutic agent for cervical cancer.

## Materials and Methods

### Cell Culture

The human HeLa cell line [HeLa 229 (ATCC^®^CCL-2.1^TM^)] used in this study was obtained from ATCC (ATCC, Manassas, VA, United States). The cells were cultured in complete medium, which is Dulbecco’s modified Eagle’s medium (DMEM) (Gibco, United States), supplemented with 10% fetal bovine serum (Merck KGaA, Germany), and antibiotics (100 U/mL penicillin and 100 μg/mL streptomycin) (Gibco, United States) and maintained under a humidified atmosphere of 37°C, 5% CO_2_. The cells were sub-cultured every 2–3 days.

### Plant Material and Extraction of *Kaempferia parviflora* Rhizomes

Fresh rhizomes of KP were harvested from the CMU-RSPG *Kaempferia* housing at Chiang Dao, Chiang Mai Province, Thailand. Voucher specimen number, R-CMUKP002, was authenticated and deposited at the Faculty of Science, Chiang Mai University, Thailand. The rhizomes of the plant were weighed, chopped, and extracted with 95% ethanol at room temperature (RT) for 3 days. Then the ethanolic extract was filtered, concentrated using a rotary evaporator, and then lyophilized. The extraction process yielded residues of 9.85% dry weight of KP rhizomes for ethanolic extraction. The crude extract was kept in an air-tight, light protected container, and stored at -20°C until used. The KP extract stock solution was freshly prepared using DMSO prior to each assay. One gram of ethanolic KP crude extract was dissolved in 1 ml of 100% DMSO to make a stock solution of 1 g/ml, and the stock was pre-diluted in medium prior to each treatment. Each experiment was performed with three independent batches of KP extract, each assayed in triplicate (*n* = 9). The final concentration of DMSO was maintained below 0.5% v/v throughout the experiment.

### Cell Viability Assay

The effect of KP on cell viability was evaluated using 3-(4,5-dimethylthiazol-2-yl)-2,5-diphenyltetrazolium bromide (MTT). The MTT assay was performed according to a previously published protocol (Mosmann, 1983). HeLa cells were seeded in 96-well plates at a density of 1 × 10^4^ cells per well for 24 h in complete medium. Cells were then treated with KP extract at various concentrations (0–1 mg/mL) or with vehicle (DMSO at 0.001–0.1%) for 24 h, then cells were exposed to the MTT reagent (0.5 mg/mL in PBS) for 2 h at 37°C, 5% CO_2_. After aspirating the culture supernatants, 200 μL of DMSO was added to each well, and the plates were incubated in the dark for 10 min. The absorbance at 590 nm was measured using a microplate reader (BioTek Instruments, United States). Cell viability assay was performed three times, and each assay was done in triplicate (*n* = 9 in three individual experiments).

### Apoptosis Assay by Flow Cytometry

Cell apoptosis was determined using FITC-annexin V (ImmunoTools, Germany) and propidium iodide (PI) (Sigma, United States). After treatment with KP extract at different concentrations (0.1, 0.3, and 0.5 mg/mL) for 6 h, cells (approximately 1 × 10^6^ cells/mL) were harvested and washed in PBS one time by centrifugation. The supernatants were discarded and cells were resuspended in 1x annexin-binding buffer. Then, annexin V and PI were added to the cell suspension, and cells were incubated at RT for 15 min. Flow cytometry was performed using a BD FACSCANTO II flow cytometer (Becton Dickinson, United States) to determine the percentage of apoptotic cell death.

### Immunofluorescence Study

HeLa cells were grown on glass cover slips for 24 h, and were then treated with KP extract at different concentrations (0.01, 0.05, and 0.1 mg/mL) for 24 h in serum-free medium. Fifteen minutes before harvesting cells, 100 ng/mL of EGF was added to KP-treated HeLa cells to activate growth and survival signaling, following which cells were fixed with 4% paraformaldehyde dissolved in PBS for 15 min. After washing three times with PBS, cells were permeabilized using 0.3% TritonX-100 in PBS for 5 min. Then cells were incubated with 1% BSA in PBS solution for 1 h, and with 1:100 of a phosphospecific (Thr 202/Tyr 204) rabbit anti-ERK1/2 antibody (Cell Signaling Technology, United States) at 4°C overnight. Cells were washed three times with PBS for 5 min each time, and incubated with a 1:500 dilution of an appropriate secondary antibody (Alexa 594-conjugated goat anti-rabbit) (Life Technologies, United States) for 2 h, in the dark, at RT. After washing three times with PBS and one time with distilled water, cells were mounted using Fluoromount-G (0100-01; SouthernBiotech, United States). Observations were performed on a fluorescence microscope, AX70 Olympus^®^, Japan, with 40x magnification, and micrographs were captured with the DP-BSW Basic Software for the DP71 microscope digital camera. To determine the ability of the KP extract to induce nuclear fragmentation, the nuclei of HeLa cell samples were stained with 5 μg/mL of Hoechst 33342 for 1 h. Cells were washed three time with PBS and one time with distilled water, and mounted as described above.

### Cell Migration Assay

A wound healing assay was performed to examine the effect of KP on cell migration. HeLa cells (0.5 × 10^6^ cells/well) were seeded and cultured in 24-well plates for 24 h. A scratch wound was made with a sterile 200 μL pipette tip. Detached cells were removed and complete medium was added. Cells were treated with DMSO as a vehicle control or KP extract (0.01, 0.05, and 0.1 mg/mL) for 40 h. Images of the scratched wounds were recorded at different time points (0, 24, and 40 h). The closing of scratched wounds is an indicator of the completion of the migration process. The migrated areas were analyzed and determined using the ImageJ software.

### Cell Invasion Assay

The effects of KP on HeLa cell invasion were determined using a Cell Culture Insert (SPL Life Sciences, South Korea). The polycarbonate invasion chambers (8 μm pore size) were coated with 15 μg of matrigel (Corning, NY, United States) per well and incubated at RT for 4 h. Cells at a density of 0.3 × 10^6^ cells per well were seeded onto the matrigel and cultured in serum-free medium for 24 h. On the next day cells in the invasion (upper) chambers were treated with different concentrations (0–0.1 mg/mL) of KP in serum-free media, and the invasion chambers were put into the (lower) wells containing DMEM with 5% FBS and incubated for 40 h. Cells were then fixed with absolute methanol for 5 min at RT and stained with 0.5% crystal violet for 30 min. After three washes with water, cells in the invasion chambers were removed with a cotton swab and the pictures of the stained cells attached at the other site of the invasion chamber were taken and analyzed with the ImageJ software.

### Gelatinase Zymography

Cells were seeded at a density of 0.3 × 10^6^ cells per well and cultured for 24 h. After KP treatment for 24 h, culture supernatants were collected and mixed with non-reducing sample buffer, and samples were separated by SDS-PAGE under cold running conditions. Following electrophoresis, the gels were washed twice in 2.5% Triton X-100 for 30 min at RT. The gels were then incubated with substrate buffer (50 mM, Tris HCL, and 10 mM CaCl_2_ pH 8) overnight. The gels were stained with 0.5% Coomassie Blue R250 in 50% methanol and 10% glacial acetic acid for 30 min, and then destaining was performed. The intensity of each band was evaluated using the ImageJ software.

### Western Blotting

For caspase and BID detection, HeLa cells were treated with 0.1, 0.3, and 0.5 mg/mL of KP extract for 6 h. For detecting the phosphorylation status of ERK1/2, Elk1, AKT, and PI3K, HeLa cells were treated with 0.01, 0.05, and 0.1 mg/mL of KP extract for 6 h. Cells were stimulated with EGF (100 ng/mL) for 15 min before harvesting cells. HeLa cell lysates were prepared by adding 1x reducing Laemmli buffer into the sample dishes. Samples were collected, heated at 95°C for 5 min, separated by SDS-PAGE, and electroblotted onto PVDF membranes (GE Healthcare Life Sciences, Germany). Membranes were blocked with 5% skim milk in TBS-T (0.02 M Tris-HCl, pH 7.6, 0.0137 M NaCl, and 0.1% (wt/v) Tween 20) at RT for 1 h. Membranes were then incubated with an appropriate primary antibody (Cell Signaling Technology, United States) at 4°C overnight. Primary antibodies used included a 1:1,000 dilution of a phosphospecific rabbit anti-PI3 kinase p85 (Tyr 458)/ p55 (Tyr 199) antibody, or a phosphospecific rabbit anti-AKT (Ser 473) (D9E) antibody, or a phosphospecific rabbit anti-ERK1/2 (Thr 202/Tyr 204) antibody, or a phosphospecific rabbit anti-Elk-1 (Ser 383), or a mouse anti-caspase-7 (C7) antibody, or a rabbit anti-caspase-9 antibody, or a rabbit anti-BID antibody, and a 1:10,000 dilution of an anti-β-actin antibody. After three washes with TBS-T, membranes were incubated with 1:5000 dilution of an appropriate horseradish peroxidase-conjugated secondary antibody (KPL, United States) for 2 h, at RT. Immune complexes were detected using enhanced chemiluminescence reagent. The intensity of the immunoreactive bands was analyzed and quantified using the ImageJ software.

### Statistical Analysis

Data were analyzed by one-way ANOVA. Data are presented as mean ± SD. In all analyses, a *p*-value (*p* < 0.05) was considered statically significant. NS indicates a non-significant difference.

## Results

### The Effects of KP on HeLa Cell Viability

**FIGURE 1:**
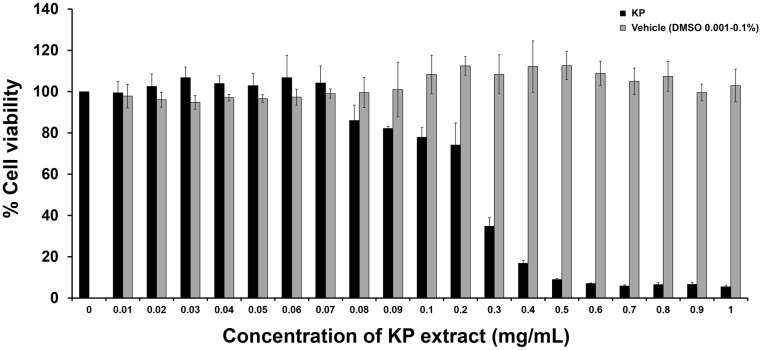
The effects of KP on HeLa cell viability. The bars indicate percent cell viability of HeLa cells treated with different concentrations of KP extract (0–1 mg/mL) for 24 h with cell viability measured by the MTT assay. Data represent mean ± SD of three independent experiments. ^∗^*p <* 0.05.

To determine the effects of KP on cell viability, cells were treated with the KP extract at different concentrations ranging from 0.01 to 1 mg/mL for 24 h. The results from MTT assay (**Figure 1**) showed that the KP extract had a strong cytotoxic effect on HeLa cells in a concentration-dependent manner. The IC_50_ value of KP extract was 0.22 mg/mL. KP extract at 0.5 mg/mL showed maximum cytotoxic effect, where approximately 90% reduction of HeLa cell viability was observed. HeLa cells treated with higher concentrations of KP extract (0.6–1 mg/mL) showed effects on cell viability similar to those treated with KP at 0.5 mg/mL. However, the KP extract at concentrations below 0.07 mg/mL did not affect HeLa cell viability. DMSO (vehicle control), at all concentrations (0.001–0.1%) relevant to the treatment group showed no apparent cytotoxicity to HeLa cells.

### The Effects of KP on Inducing Cell Apoptosis

**FIGURE 2:**
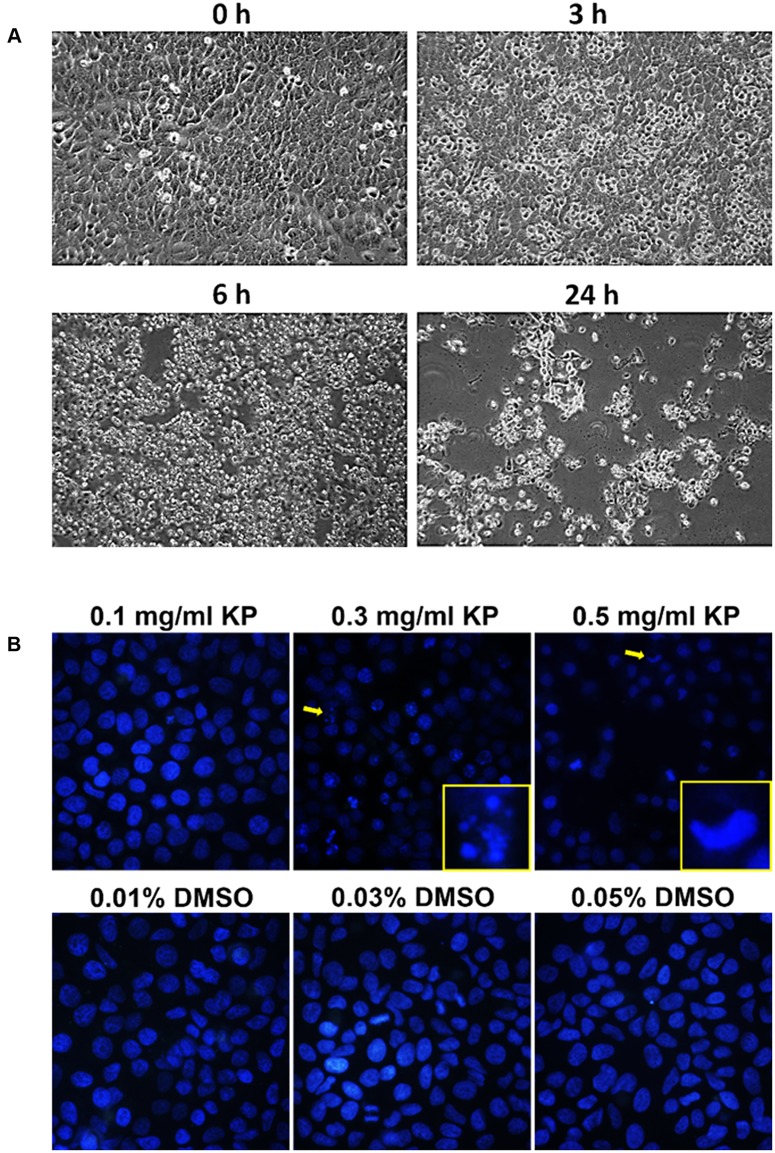
Morphological changes and nuclear fragmentation of HeLa cells exposed to KP extract. **(A)** Phase-contrast images of HeLa cells treated with 0.5 mg/mL of KP extract taken at different time points (0, 3, 6, and 24 h). **(B)** The nuclei of HeLa cells treated with KP extract at different concentrations (0.1–0.5 mg/mL) or with DMSO (0.01–0.05%), stained with Hoechst33342, and visualized using a fluorescent microscope. Arrows indicate cells with nuclear fragmentation or nuclear deformity, and magnified views of cells indicated with arrows are shown at the bottom right corners. Data are representative of three replicates.

**FIGURE 3:**
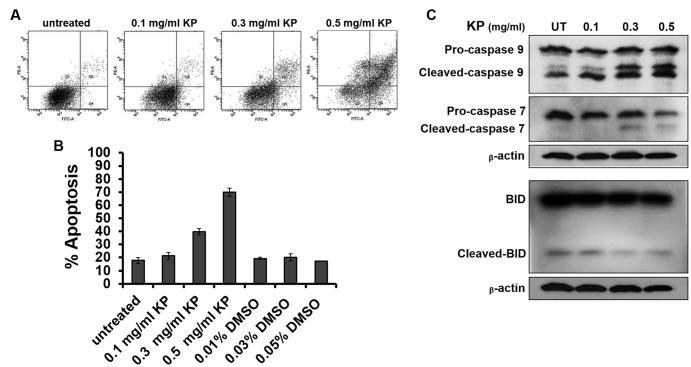
The effects of KP on inducing HeLa cell apoptosis. **(A)** Representative figures from flow cytometry showing HeLa cells undergoing apoptotic cell death upon incubation with different concentrations (0–0.5 mg/mL) of KP extract for 6 h. **(B)** Quantitative analysis of percentage cell apoptosis from flow cytometry. Data are representative of three replicates and are expressed as mean ± SD. **(C)** A representative western blot of caspase 9, caspase 7, and BID from HeLa cells treated with different concentrations (0–0.5 mg/mL) of KP extract for 6 h. Two immunoreactive bands of cleaved-caspase 9 indicate a p35 subunit and a p37 subunit of active caspase 9. Beta-actin was used as a loading control.

To investigate whether KP induces apoptotic cell death, we first observed for morphological aberrations of HeLa cells in response to KP extract exposure. Since KP extract at 0.5 mg/mL showed maximum cytotoxic effect, HeLa cells were exposed to 0.5 mg/mL of KP extract and phase-contrast images of cells were taken at different time points (0–24 h). Data showed that KP extract caused HeLa cell morphological changes in a time-dependent manner. **Figure 2A** shows that at 0 h of KP treatment, HeLa cells were observed to exhibit normal morphology of epithelial cells with discrete cell-cell-contact, and most cells were tightly attached to the surface of the dish. After 3 h of treatment, even though cells were still attached to the dish, the vast majority of cells showed distinct morphological changes. Those cells changed from the normal characteristic elongated shape and become spherical. After 6 h, all cells exhibited spherical morphology, and cell detachment from the dish was observed in some areas. Eventually, most cells detached from the surface after 24 h of KP treatment, and the remaining cells with aberrant morphology were likely to be dead or dying cells. Since one of the characteristics of apoptotic cell death is nuclear fragmentation, we performed nuclear staining using Hoechst 33342 of HeLa cells treated with KP extract at various concentrations (0.1, 0.3, and 0.5 mg/mL) for 6 h. We found that KP extract at potent cytotoxic concentrations (0.3 and 0.5 mg/mL) induced nuclear deformity and nuclear fragmentation of HeLa cells after 6 h of incubation, and these effects were independent of DMSO used as the vehicle control (**Figure 2B**). Based on these observations, we hypothesized that KP has the potential ability to induce programmed cell death. We therefore performed flow cytometry to determine whether KP induces HeLa cell apoptosis. As expected, KP extract induced apoptotic cell death in a concentration-dependent manner. We found that HeLa cell apoptosis was significantly increased to 39.8 ± 2.40% for cells treated with 0.3 mg/mL KP and to 69.85 ± 3.04% for cells treated with 0.5 mg/mL of KP extract, whereas DMSO at all concentrations relevant to those present in KP treatment groups did not induce apoptosis in HeLa cells (**Figures 3A,B**). We further confirmed the effects of KP on inducing apoptosis in HeLa cells by performing western blot analysis to determine the activation of caspase 9, caspase 7, and the pro-apoptotic protein BID, using specific antibodies that can recognize both full length and cleaved forms of the proteins. The results showed that the KP extract induced cleavage of caspase 7 and 9 in a concentration-dependent manner, however, the extract at all concentrations tested did not increase the cleaved form of BID (**Figure 3C**).

### The Effects of KP on Inhibiting Cell Migration and Invasion

**FIGURE 4:**
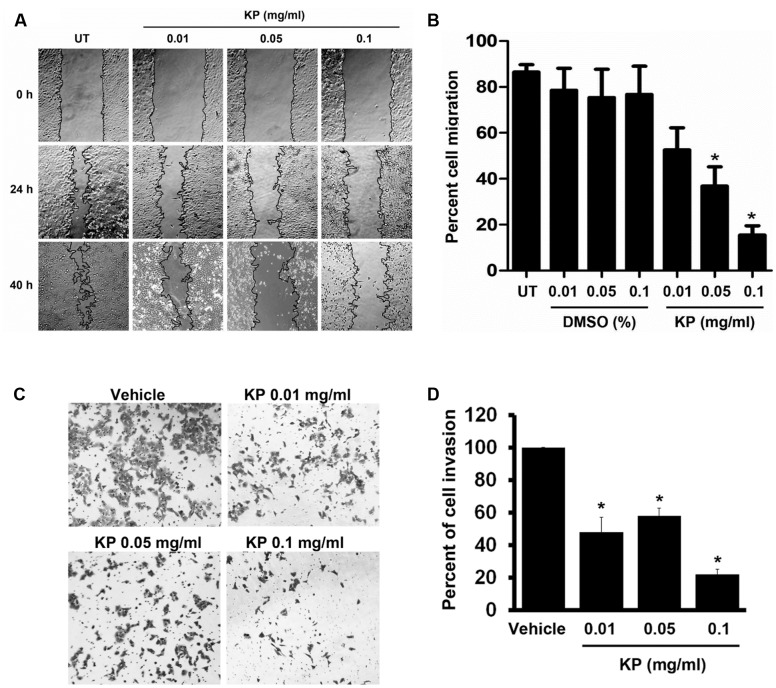
The effects of KP on HeLa cell migration and invasion. **(A)** Scratch wounds of monolayers HeLa cells treated with KP extract (0.01, 0.05, and 0.1 mg/mL) for 40 h. Cell migration was monitored with 4x magnification, and phase-contrast images of cell migration were taken at the time of the scratch and at 24 and 40 h post-scratch. **(B)** Quantitative analysis of cell migration into the scratch wound at 40 h post-scratch. Data are expressed as mean ± SD. Asterisks indicate significantly different from the control groups (untreated groups) (^∗^*p <* 0.05). **(C)** Representative images of HeLa cell invasion treated with KP extract (0.01, 0.05, and 0.1 mg/mL) and examined by the Transwell invasion assay. Vehicle is the control group where cells were treated with the highest concentration of DMSO (0.01%) which corresponded to the concentration present in 0.1 mg/mL of KP. **(D)** Quantitative analysis of percent of cell invasion in KP-treated cells compared to the vehicle control. Data are representative of three replicates and are expressed as mean ± SD. ^∗^*p <* 0.05 compared with the vehicle control.

One of the important characteristics of malignant tumors is enhanced cell migration. We determined whether the KP extract can inhibit migration of HeLa cells by performing a wound healing assay. Results showed that the KP extract significantly suppressed the migration of HeLa cells at both 24 and 40 h, and the suppression was seen in a concentration-dependent manner (**Figure 4A**). Compared to untreated HeLa cells which exhibited 86.4 ± 77.22% cell migration at 40 h, cells treated with 0.01, 0.05, and 0.1 mg/mL of KP extract showed decreases in the percent cell migration to approximately 52.53 ± 21%, 36.77 ± 18.8%, and 15.46 ± 9.13%, respectively (**Figure 4B**). The percent cell migration in cells treated with 0.01, 0.05, and 0.1% of DMSO (vehicle control) was 78.53 ± 21.46%, 75.35 ± 27.71%, and 76.76 ± 27.58%, respectively. Based on the observation that KP extract significantly inhibited HeLa cell migration, it is reasonable to hypothesize that KP also inhibits cell invasion and so a cell invasion test was performed using the Transwell invasion assay. We found that KP extract significantly reduced cell movement through the matrigel-coated chamber in a concentration-dependent manner (**Figure 4C**). The percentage of cell invasion compared to that of the vehicle control (0.1% DMSO) was reduced to 47.79 ± 9.21%, 57.83 ± 4.97%, and 21.88 ± 3.21% for HeLa cells treated with 0.01, 0.05, and 0.1 mg/mL of KP extract, respectively (**Figure 4D**).

### The Effect of KP on Inhibiting Metalloproteinase-2 Activity

**FIGURE 5:**
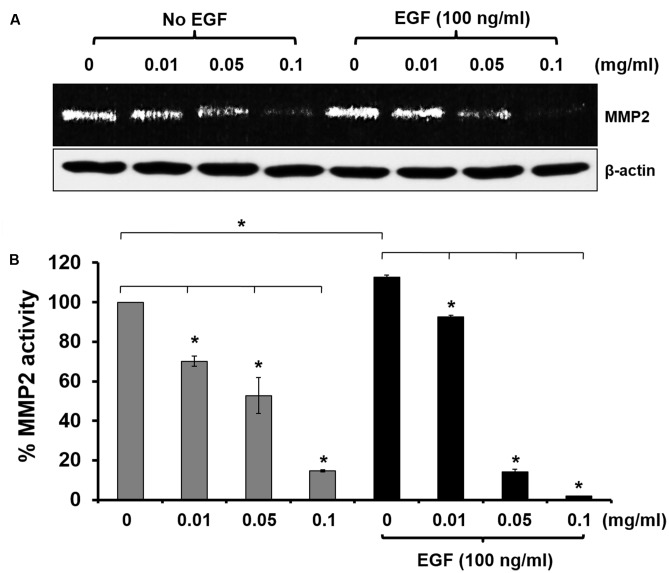
Effects of KP extract on suppressing MMP-2 activity. **(A)** Zymographic analysis for MMP-2 activity in HeLa cells treated with KP extract at various concentrations (0–0.1 mg/mL) with or without the presence of 100 ng/mL of EGF. Western blot for beta actin was used as a loading control. **(B)** Quantitative analysis of MMP-2 level using ImageJ software. Beta actin from the western blot was used as an internal control for normalization. Data are expressed as mean ± SD. ^∗^*p <* 0.05 compared with untreated cells.

Since we observed that KP inhibited HeLa cell migration and invasion, we then tested our hypothesis that KP may suppress the activity of MMP-2, which is a major metalloproteinase expressed by HeLa cells. Data from gelatinase zymography shown in **Figures 5A,B** demonstrated that the KP extract significantly reduced MMP-2 activity in a concentration-dependent manner. The immunoreactive bands of actin detected by western blot showed the approximately equal loading in each group. Approximately 30, 50, and 90% inhibition of MMP-2 activity was observed in HeLa cells treated with 0.01, 0.05, and 0.1 mg/mL of KP extract, respectively. When the treatment was performed in the presence of EGF at 100 ng/mL, we found that EGF slightly increased the production of MMP-2 by HeLa cells. Nevertheless, KP extract was still able to potently inhibit MMP-2 activity in a concentration-dependent manner.

### The Effects of KP on Suppressing Growth and Survival Signal Transduction Pathways

**FIGURE 6:**
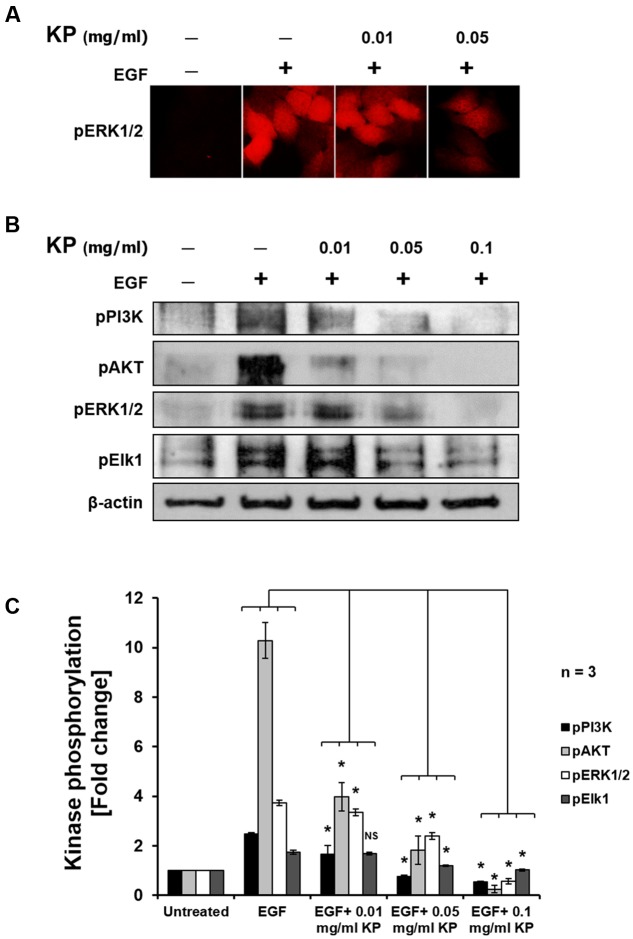
The effects of KP extract on suppressing growth and survival signal transduction pathways. **(A)** Immunofluorescence of pERK1/2 in HeLa cells treated with KP extract. **(B)** Western blot showing immunoreactive bands of pPI3K, pAKT, pERK1/2, pElk1, and β-actin of HeLa cells stimulated with 100 ng/mL EGF and treated with different concentrations of KP extract. **(C)** Quantitative analysis of phosphorylation status of PI3K, AKT, ERK1/2, and Elk1 of HeLa cells treated with 100 ng/mL EGF and different concentrations of KP extract. Beta actin was used as an internal control and for normalization. Data are representative of three independent replicates and are expressed as mean ± SD. ^∗^*p <* 0.05 compared with untreated cells.

To further investigate the effects of KP extract on growth of HeLa cells, the phosphorylation status of several major protein kinases, including PI3K, AKT, ERK1/2, and Elk1, in response to KP extract treatment was determined. We initially undertook immunoflourescent staining of an important member of the MAPK signaling pathway, pERK1/2, in HeLa cells treated with KP extract at different concentrations, to examine whether KP extract can inhibit growth factor mediated signaling. Results from the immunofluorescence study showed that serum-starved HeLa cells showed a very low basal level of ERK1/2 phosphorylation (**Figure 6A**; additional images representing more cells are shown in **Supplementary Figure S1**). When EGF at 100 ng/mL was added to serum-starved HeLa cells, phosphorylation of ERK1/2 was markedly increased. However, EGF-treated cells exposed to KP extract at 0.01 and 0.05 mg/mL showed obvious reductions of ERK1/2 phosphorylation. Based on these results, we further defined whether KP extract can affect phosphorylation status of other crucial players in growth and survival signal transduction pathways. We therefore undertook western blot analysis of a number of signal transduction kinases, and the results showed that KP extract could effectively suppress the EGF-dependent phosphorylation of PI3K, AKT, ERK1/2, and Elk1 in a concentration-dependent manner. **Figures 6B,C** shows that HeLa cells stimulated with EGF alone for 15 min exhibited an increase in phosphorylation of PI3K (2.82 ± 0.06 fold), AKT (10.28 ± 0.72 fold), ERK1/2 (4.27 ± 0.11 fold), and Elk1 (1.74 ± 0.08 fold). Interestingly, pretreatment of HeLa cells with 0.01 mg/mL of KP extract for 6 h before the addition of EGF dramatically reduced stimulated phosphorylation of PI3K to 1.64 ± 0.36 fold, AKT to 3.98 ± 0.57 fold, ERK1/2 to 2.84 ± 0.12 fold, and Elk1 to 1.67 ± 0.05 fold. Moreover, EGF-stimulated cells treated with 0.05 mg/mL KP extract significantly reduced phosphorylation of PI3K to 0.74 ± 0.06 fold, AKT to 1.81 ± 0.57 fold, ERK1/2 to 2.24 ± 0.14 fold, and Elk1 to 1.19 ± 0.02 fold. Finally, EGF-treated cells treated with 0.1 mg/mL KP extract significantly reduced phosphorylation of PI3K to 0.54 ± 0.03 fold, AKT to 0.24 ± 0.14 fold, ERK1/2 to 0.56 ± 0.11 fold, and Elk1 to 1.01 ± 0.04 fold. Beta actin was used as an internal control and for normalization. The immunoreactive bands of actin indicated approximately equal protein loading in each well.

## Discussion

Although the 5-year overall survival for women diagnosed with early-stage cervical cancer is more than 90% (Quinn et al., 2006), cervical cancer is currently the fourth most common cancer in women worldwide (Zigras et al., 2017). Conventionally, chemotherapy, radiation, and surgery are common treatments for all patients, however, most of these conventional therapeutic strategies have severe adverse effects to the patients (Petignat and Roy, 2007).

Alternative medicines have emerged as one of potential treatments with less side effects and a more affordable cost (Wang et al., 2013), and in particular medicinal herbs are major sources of anti-cancer drug discovery and development (Desai et al., 2008). In the current study, we investigated KP, which has been reported to possess anti-cancer properties against a number of different cancer cell lines (Banjerdpongchai et al., 2008). However, evidence for mechanism of action of KP is very limited, and in particular data about its roles in modulating molecular signal transduction in cervical cancer is not available.

In this study, we investigated the anti-cancer activities of KP against the cervical cancer cell line HeLa. We found that KP extract reduced cervical cancer cell viability in a concentration dependent manner. Our results are consistent with previous studies showing that KP exhibited cytotoxicity to human colorectal carcinoma (HCT-15) cells and to human leukemic (U937) cells (Banjerdpongchai et al., 2008, 2009). Moreover, it has been reported that 5,7,4-trimethoxyflavone (KP.8.10), which is one of the major constituents of KP, inhibited proliferation of human cholangiocarcinoma cell lines (HuCCA-1 and RMCCA-1) (Leardkamolkarn et al., 2009). Results from our study highlight the genuine cytotoxic activity of KP against different cancers.

Based on the results from the MTT assay, we further proved that KP reduces viability of HeLa cells via the induction of programmed cell death. We first monitored changes in morphology of HeLa cells exposed to a cytotoxic concentration of KP extract (0.5 mg/mL) at different time points and observed that KP extract gradually induced morphological changes over time and eventually caused HeLa cell detachment from the surface of the culture dish at 24 h of incubation. The rounding and detachment of cells is one of the classic hallmarks of programmed cell death. To further verify our hypothesis that KP induces apoptosis in HeLa cells, we stained KP-treated cells with annexin V/PI and performed flow cytometry analysis. We found that KP significantly induced apoptosis in HeLa cells after 6 h of incubation and this induction was in a concentration-dependent manner. Our observations are similar to those reported in 2008 where an ethanolic extract of KP significantly induced apoptosis in HL-60 cells as evaluated by flow cytometry (Banjerdpongchai et al., 2008). Consistent with our results obtained from flow cytometry, when KP-treated HeLa cells were stained with Hoechst 33342, the aberration of the nuclei was observed. Specifically, we clearly showed that KP induced nuclear deformity and nuclear fragmentation in HeLa cells. Nuclear fragmentation is known to be one of major characteristics of cells undergoing apoptosis where the nuclear lamina is destabilized by active caspases (Elmore, 2007). Therefore, nuclear fragmentation observed in our study confirms that KP kills HeLa cells via the induction of apoptosis.

We next defined which specific apoptotic signaling pathways are activated in response to KP treatment. Western blot analysis clearly demonstrated that KP induced caspase 7 and 9 activation in a concentration-dependent manner, indicating that the intrinsic apoptotic pathway is stimulated in response to KP extract exposure. However, KP did not induce any change in the level of full-length or cleaved BID indicating that caspase 8 in the Fas signaling pathway (extrinsic pathway) was not activated in HeLa cells treated with KP extract. Hence, we proposed that the activation of the intrinsic apoptotic pathway is an underlying mechanism of action of KP in inducing HeLa cell death.

The observation that KP extract at cytotoxic concentrations could potently induce apoptosis in HeLa cells is interesting. However, we additionally explored the effects of KP at non-cytotoxic concentrations in modulating growth and survival signaling of cervical cancer cells, and major signal transduction pathways relevant to cell growth and cell survival including the MAPK and PI3K/AKT pathways were investigated and in particular the inhibitory effects of KP on growth and survival signaling over the influence of EGF was explored.

EGF is a growth factor that stimulates cell growth, proliferation, and differentiation of target cells by binding and activating the specific EGFR receptor (She et al., 2005). The activation of the tyrosine kinase EGFR leads to further stimulation of several different downstream signal transduction cascades, including the MAPK and PI3K/AKT pathways, which eventually lead to changes in cellular activity (She et al., 2005). One of important events after EGF activation is an increase in cell division which is a hallmark of tumors (Normanno et al., 2006). Furthermore, constitutive activation of the PI3K/AKT pathway in most cancers is typical, and has been shown to promote cancer cell survival (Zhang et al., 2015). Therefore, agents or therapeutic strategies that can adequately inhibit the over activation of EGF or the downstream effectors in the signaling cascade would be interesting targets for developing as effective anti-cancer therapies (Harari, 2004). We hypothesized that KP extract at low and non-cytotoxic concentrations may suppress the activation of the MAPK and AKT signaling.

The results confirmed our hypothesis since KP extract significantly suppressed phosphorylation of PI3K, AKT, ERK1/2, and Elk1. The reduction in phosphorylation status observed in PI3K and AKT suggests that survival signaling in HeLa cells is interrupted even though EGF was present. Similar observations were seen in ERK1/2 in cells exposed to KP extract at non-cytotoxic concentrations, suggesting that cell growth and proliferation signaling in HeLa cells is suppressed. MAPKs are among the central elements that transduce extracellular stimuli into cellular responses, and are known to play a crucial role in cell growth (Seger and Krebs, 1995). In general, when ERK1/2 is activated by phosphorylation, it translocates into the nucleus and further phosphorylates and activates several different transcription factors involved in cell cycle progression (Chambard et al., 2007). One of those pERK1/2 targets is Elk1 (Mebratu and Tesfaigzi, 2009). A noticeable decrease in Elk1 phosphorylation in KP-treated HeLa cells along with that of ERK1/2 verifies that KP has a potent property to inhibit EGF-dependent activation of HeLa cell growth and survival signaling. It is reasonable to conclude that KP extract can not only induce cell apoptosis but also suppresses growth and survival of cervical cancer cells.

Besides effects of KP extract on growth and survival signaling, there are other interesting aspects to be explored. The observation that phosphorylation of Elk1 is significantly reduced after incubation with KP extract led us to believe that KP also has a role in regulating the remodeling of the extracellular matrix of HeLa cells via interfering with the expression of metalloproteinase 2. It has been reported that the activated Elk1 controls the expression of molecules engaged in the proteolysis of the extracellular matrix, such as MMP-2 and MMP-9 (Choi et al., 2011). Consequently, Elk1 is able to control cell migration and invasion (Odrowaz and Sharrocks, 2012). Interestingly, consistent with previous reports, we found that KP inhibited the activity of MMP-2 in a concentration dependent manner and this effect was still observed in HeLa cells treated with KP extract in the presence of EGF. This means that KP has a potent effect over the effect of EGF to suppress MMP-2 production. MMP expression has been shown to be linked to tumor invasion in many different tumors (Liotta et al., 1980; Cottam et al., 1992; Garzetti et al., 1995; Fidler, 1997; Fishman et al., 1997; Gohji et al., 1998; John and Tuszynski, 2001). In addition, many clinical studies have emphasized the association of MMP expression with progression of cervical cancer (Zhou et al., 2002; Asha Nair et al., 2003), and many types of human tumor have been reported to be associated with increased expression of MMP-2 (Di Nezza et al., 2002; Sato et al., 2004; Berube et al., 2005).

Our current study reporting that KP extract can effectively reduce MMP-2 activity provides valuable information to support that KP may inhibit migration and invasion of cervical cancer cells. Therefore, we performed cell migration and invasion assays and found that KP extract at non-cytotoxic concentrations significantly inhibited migration and invasion of HeLa cells in a concentration-dependent manner. This observation elicits a role for KP extract in suppressing remodeling of the extracellular matrix and inhibiting migration and invasion. This statement is supported by our finding that KP inhibited the PI3K/AKT signaling which is a signal transduction pathway reported to be associated with cell motility and invasion (Zhou and Wong, 2006). Since KP extract at the concentrations that did not kill cells could successfully suppress migration and invasion of HeLa cells, we strongly believe that KP possesses its authentic anti-cancer property against cervical cancer, at least in part, through inhibition of migration and invasion.

## Conclusion

This study presents accumulated evidence that *Kaempferia parviflora* extract possesses anti-cancer properties including the suppression of growth and survival signaling pathways, inhibition of metalloproteinase 2 activity, inhibition of cell migration and invasion, and induction of apoptosis in the HeLa cervical cancer cell line. Our report strongly suggests that *Kaempferia parviflora* contains active compounds which may directly or indirectly inhibit EGF-dependent signal transduction pathways and subsequently suppress tumor progression and induce cancer cell death. Identification of potential active KP compounds along with determination of their interaction with EGF receptor or specific downstream effectors is worth further investigation since the obtained information would be beneficial for verifying that *Kaempferia parviflora* can be used as a valid chemopreventive and chemotherapeutic agent in cervical cancer treatment.

## Author Contributions

The experiments, data analysis, and manuscript writing were conducted by SP, SS, MNT, PM, and NW. DS provided vital technical support for the project and proofread the manuscript. WN founded the research project, designed the experiments, writing the manuscript, and contributed to the funding of the project.

## Conflict of Interest Statement

The authors declare that the research was conducted in the absence of any commercial or financial relationships that could be construed as a potential conflict of interest. The reviewer JB and handling Editor declared their shared affiliation.

## Supplementary Material

**FIGURE S1 |** Immunofluorescence of pERK1/2 in HeLa cells treated with KP extract at different concentrations (10 and 50 μg/mL) with or without the presence of 100 ng/mL EGF.

## References

[B1] Asha Nair S. Karunagaran D. Nair M. B. Sudhakaran P. R. (2003). Changes in matrix metalloproteinases and their endogenous inhibitors during tumor progression in the uterine cervix. *J. Cancer Res. Clin. Oncol.* 129 123–131. 10.1007/s00432-002-0411-9 12669237 PMC12161933

[B2] Banjerdpongchai R. Chanwikruy Y. Rattanapanone V. Sripanidkulchai B. (2009). Induction of apoptosis in the human Leukemic U937 cell line by *Kaempferia parviflora* Wall.ex.Baker extract and effects of paclitaxel and camptothecin. *Asian Pac. J. Cancer Prev.* 10 1137–1140. 20192599

[B3] Banjerdpongchai R. Suwannachot K. Rattanapanone V. Sripanidkulchai B. (2008). Ethanolic rhizome extract from *Kaempferia parviflora* Wall. ex. Baker induces apoptosis in HL-60 cells. *Asian Pac. J. Cancer Prev.* 9 595–600. 19256745

[B4] Berube M. Deschambeault A. Boucher M. Germain L. Petitclerc E. Guerin S. L. (2005). MMP-2 expression in uveal melanoma: differential activation status dictated by the cellular environment. *Mol. Vis.* 11 1101–1111. 16379022

[B5] Canavan T. P. Doshi N. R. (2000). Cervical cancer. *Am. Fam. Physician* 61 1369–1376. 10735343

[B6] Cantley L. C. (2002). The phosphoinositide 3-kinase pathway. *Science* 296 1655–1657. 10.1126/science.296.5573.1655 12040186

[B7] Chakravarti A. Chakladar A. Delaney M. A. Latham D. E. Loeffler J. S. (2002). The epidermal growth factor receptor pathway mediates resistance to sequential administration of radiation and chemotherapy in primary human glioblastoma cells in a RAS-dependent manner. *Cancer Res.* 62 4307–4315. 12154034

[B8] Chambard J.-C. Lefloch R. Pouysségur J. Lenormand P. (2007). ERK implication in cell cycle regulation. *Biochim. Biophys. Acta* 1773 1299–1310. 10.1016/j.bbamcr.2006.11.010 17188374

[B9] Choi B. D. Jeong S. J. Wang G. Park J. J. Lim D. S. Kim B. H. (2011). Secretory leukocyte protease inhibitor is associated with MMP-2 and MMP-9 to promote migration and invasion in SNU638 gastric cancer cells. *Int. J. Mol. Med.* 28 527–534. 10.3892/ijmm.2011.726 21687932

[B10] Cottam D. W. Rennie I. G. Woods K. Parsons M. A. Bunning R. A. Rees R. C. (1992). Gelatinolytic metalloproteinase secretion patterns in ocular melanoma. *Invest. Ophthalmol. Vis. Sci.* 33 1923–1927. 1316333

[B11] Denny L. Quinn M. Sankaranarayanan R. (2006). Chapter 8: screening for cervical cancer in developing countries. *Vaccine* 24(Suppl. 3), S71–S77. 10.1016/j.vaccine.2006.05.121 16950020

[B12] Desai A. G. Qazi G. N. Ganju R. K. El-Tamer M. Singh J. Saxena A. K. (2008). Medicinal plants and cancer chemoprevention. *Curr. Drug Metab.* 9 581–591. 10.2174/138920008785821657 18781909 PMC4160808

[B13] Di Nezza L. A. Misajon A. Zhang J. Jobling T. Quinn M. A. Östör A. G. (2002). Presence of active gelatinases in endometrial carcinoma and correlation of matrix metalloproteinase expression with increasing tumor grade and invasion. *Cancer* 94 1466–1475. 10.1002/cncr.10355 11920503

[B14] Elmore S. (2007). Apoptosis: a review of programmed cell death. *Toxicol. Pathol.* 35 495–516. 10.1080/01926230701320337 17562483 PMC2117903

[B15] Fidler I. J. (1997). “Molecular biology of cancer: invasion and metastasis,” in *Cancer: Principles and Practice of Oncology*, 5th Edn, eds Vita V. T. De Hellman S. Rosenberg S. A. (Philadelphia, PA: Lippincott-Raven), 135–152.

[B16] Fishman D. A. Bafetti L. M. Banionis S. Kearns A. S. Chilukuri K. Stack M. S. (1997). Production of extracellular matrix-degrading proteinases by primary cultures of human epithelial ovarian carcinoma cells. *Cancer* 80 1457–1463. 10.1002/(SICI)1097-0142(19971015)80:8<1457::AID-CNCR13>3.0.CO;2-4 9338470

[B17] Garzetti G. G. Ciavattini A. Lucarini G. Goteri G. de e Nictolis M. Garbisa S. (1995). Tissue and serum metalloproteinase (MMP-2) expression in advanced ovarian serous cystoadenocarcinomas: clinical and prognostic implications. *Anticancer Res.* 15 2799–2804. 8669868

[B18] Geisler J. P. Swathirajan J. Wood K. L. Manahan K. J. (2012). Treatment of advanced or recurrent cervical cancer with cisplatin or cisplatin containing regimens: a cost effective analysis. *J. Cancer* 3 454–458. 10.7150/jca.4807 23236342 PMC3520020

[B19] Gohji K. Fujimoto N. Hara I. Fujii A. Gotoh A. Okada H. (1998). Serum matrix metalloproteinase-2 and its density in men with prostate cancer as a new predictor of disease extension. *Int. J. Cancer* 79 96–101. 10.1002/(SICI)1097-0215(19980220)79:1<96::AID-IJC18>3.0.CO;2-F 9495366

[B20] Hanahan D. Weinberg R. A. (2011). Hallmarks of cancer: the next generation. *Cell* 144 646–674. 10.1016/j.cell.2011.02.013 21376230

[B21] Harari P. M. (2004). Epidermal growth factor receptor inhibition strategies in oncology. *Endocr. Relat. Cancer* 11 689–708. 10.1677/erc.1.00600 15613446

[B22] Huang H. S. Nagane M. Klingbeil C. K. Lin H. Nishikawa R. Ji X. D. (1997). The enhanced tumorigenic activity of a mutant epidermal growth factor receptor common in human cancers is mediated by threshold levels of constitutive tyrosine phosphorylation and unattenuated signaling. *J. Biol. Chem.* 272 2927–2935. 10.1074/jbc.272.5.2927 9006938

[B23] John A. Tuszynski G. (2001). The role of matrix metalloproteinases in tumor angiogenesis and tumor metastasis. *Pathol. Oncol. Res.* 7 14–23. 10.1007/BF03032599 11349215

[B24] Leardkamolkarn V. Tiamyuyen S. Sripanidkulchai B. O. (2009). Pharmacological activity of *Kaempferia parviflora* extract against human bile duct cancer cell lines. *Asian Pac. J. Cancer Prev.* 10 695–698. 19827897

[B25] Liotta L. A. Tryggvason K. Garbisa S. Hart I. Foltz C. M. Shafie S. (1980). Metastatic potential correlates with enzymatic degradation of basement membrane collagen. *Nature* 284 67–68. 10.1038/284067a0 6243750

[B26] Liu H. Wang H. Li C. Zhang T. Meng X. Zhang Y. (2016). Spheres from cervical cancer cells display stemness and cancer drug resistance. *Oncol. Lett.* 12 2184–2188. 10.3892/ol.2016.4893 27602161 PMC4998566

[B27] Margolis B. (1992). Proteins with SH2 domains: transducers in the tyrosine kinase signaling pathway. *Cell Growth Diff.* 3 73–80. 1534689

[B28] McGuire S. (2016). World cancer report 2014. Geneva, Switzerland: world health organization, international agency for research on cancer, WHO press, 2015. *Adv. Nutr.* 7 418–419. 10.3945/an.116.012211 26980827 PMC4785485

[B29] Mebratu Y. Tesfaigzi Y. (2009). How ERK1/2 activation controls cell proliferation and cell death is subcellular localization the answer? *Cell Cycle* 8 1168–1175. 19282669 10.4161/cc.8.8.8147 PMC2728430

[B30] Moody C. A. Laimins L. A. (2010). Human papillomavirus oncoproteins: pathways to transformation. *Nat. Rev. Cancer* 10 550–560. 10.1038/nrc2886 20592731

[B31] Moscatello D. K. Holgado-Madruga M. Emlet D. R. Montgomery R. B. Wong A. J. (1998). Constitutive activation of phosphatidylinositol 3-kinase by a naturally occurring mutant epidermal growth factor receptor. *J. Biol. Chem.* 273 200–206. 10.1074/jbc.273.1.200 9417065

[B32] Mosmann T. (1983). Rapid colorimetric assay for cellular growth and survival: application to proliferation and cytotoxicity assays. *J. Immunol. Methods* 65 55–63. 10.1016/0022-1759(83)90303-4 6606682

[B33] Normanno N. De Luca A. Bianco C. Strizzi L. Mancino M. Maiello M. R. (2006). Epidermal growth factor receptor (EGFR) signaling in cancer. *Gene* 366 2–16. 10.1016/j.gene.2005.10.018 16377102

[B34] Odrowaz Z. Sharrocks A. D. (2012). The ETS transcription factors ELK1 and GABPA regulate different gene networks to control MCF10A breast epithelial cell migration. *PLOS ONE* 7:e49892 10.1371/journal.pone.0049892 PMC3527487 23284628

[B35] Olayioye M. A. Neve R. M. Lane H. A. Hynes N. E. (2000). The ErbB signaling network: receptor heterodimerization in development and cancer. *EMBO J.* 19 3159–3167. 10.1093/emboj/19.13.3159 10880430 PMC313958

[B36] Patanasethanont D. Nagai J. Matsuura C. Fukui K. Sutthanut K. Sripanidkulchai B. O. (2007). Modulation of function of multidrug resistance associated-proteins by *Kaempferia parviflora* extracts and their components. *Eur. J. Pharmacol.* 566 67–74. 10.1016/j.ejphar.2007.04.001 17481606

[B37] Petignat P. Roy M. (2007). Diagnosis and management of cervical cancer. *BMJ* 335 765–768. 10.1136/bmj.39337.615197.80 17932207 PMC2018789

[B38] Quinn M. A. Benedet J. L. Odicino F. Maisonneuve P. Beller U. Creasman W. T. (2006). Carcinoma of the cervix uteri. FIGO 26th annual report on the results of treatment in gynecological cancer. *Int. J. Gynaecol. Obstet.* 95(Suppl. 1), S43–S103. 10.1016/S0020-7292(06)60030-1 17161167

[B39] Roomi M. W. Monterrey J. C. Kalinovsky T. Rath M. Niedzwiecki A. (2010). In vitro modulation of MMP-2 and MMP-9 in human cervical and ovarian cancer cell lines by cytokines, inducers and inhibitors. *Oncol. Rep.* 23 605–614. 10.3892/or_00000675 20126997

[B40] Safaeian M. Solomon D. (2007). Cervical cancer prevention - cervical screening: science in evolution. *Obstet. Gynecol. Clin. North Am.* 34 739–799. 10.1016/j.ogc.2007.09.004 18061867 PMC2762353

[B41] Saokaew S. Wilairat P. Raktanyakan P. Dilokthornsakul P. Dhippayom T. Kongkaew C. (2016). Clinical effects of Krachaidum (*Kaempferia parviflora*): a systematic review. *J. Evid. Based Complement. Altern. Med.* 10.1177/2156587216669628 [Epub ahead of print]. PMC5871153 27694558

[B42] Saslow D. Castle P. E. Cox J. T. Davey D. D. Einstein M. H. Ferris D. G. (2007). American Cancer Society Guideline for human papillomavirus (HPV) vaccine use to prevent cervical cancer and its precursors. *CA Cancer J. Clin.* 57 7–28. 10.3322/canjclin.57.1.7 17237032

[B43] Sato T. Sakai T. Noguchi Y. Takita M. Hirakawa S. Ito A. (2004). Tumor-stromal cell contact promotes invasion of human uterine cervical carcinoma cells by augmenting the expression and activation of stromal matrix metalloproteinases. *Gynecol. Oncol.* 92 47–56. 10.1016/j.ygyno.2003.09.012 14751137

[B44] Seger R. Krebs E. G. (1995). The MAPK signaling cascade. *FASEB J.* 9 726–735. 7601337

[B45] She Q.-B. Solit D. B. Ye Q. O’Reilly K. E. Lobo J. Rosen N. (2005). The BAD protein integrates survival signaling by EGFR/MAPK and PI3K/Akt kinase pathways in PTEN-deficient tumor cells. *Cancer Cell* 8 287–297. 10.1016/j.ccr.2005.09.006 16226704 PMC3203692

[B46] Siegel R. L. Miller K. D. Jemal A. (2016). Cancer statistics, 2016. *CA Cancer J. Clin.* 66 7–30. 10.3322/caac.21332 26742998

[B47] Stetler-Stevenson W. G. (2001). The role of matrix metalloproteinases in tumor invasion, metastasis, and angiogenesis. *Surg. Oncol. Clin. N. Am.* 10 383–392. 11382593

[B48] Subramanian S. Trogdon J. Ekwueme D. U. Gardner J. G. Whitmire J. T. Rao C. (2010). Cost of cervical cancer treatment: implications for providing coverage to low-income women under the Medicaid expansion for cancer care. *Womens Health Issues* 20 400–405. 10.1016/j.whi.2010.07.002 21050999

[B49] Surviladze Z. Sterk R. T. DeHaro S. A. Ozbun M. A. (2013). Cellular entry of human papillomavirus type 16 involves activation of the phosphatidylinositol 3-Kinase/Akt/mTOR pathway and inhibition of autophagy. *J. Virol.* 87 2508–2517. 10.1128/jvi.02319-12 23255786 PMC3571372

[B50] Vivanco I. Sawyers C. L. (2002). The phosphatidylinositol 3-Kinase AKT pathway in human cancer. *Nat. Rev. Cancer* 2 489–501. 10.1038/nrc839 12094235

[B51] Wang S. J. Zheng C. J. Peng C. Zhang H. Jiang Y. P. Han T. (2013). Plants and cervical cancer: an overview. *Expert Opin. Investig. Drugs* 22 1133–1156. 10.1517/13543784.2013.811486 23789984

[B52] Yarden Y. Sliwkowski M. X. (2001). Untangling the ErbB signalling network. *Nat. Rev. Mol. Cell Biol.* 2 127–137. 10.1038/35052073 11252954

[B53] Yin S.-Y. Wei W.-C. Jian F.-Y. Yang N.-S. (2013). therapeutic applications of herbal medicines for cancer patients. *Evid. Based Complement. Altern. Med.* 2013:302426 10.1155/2013/302426 PMC3727181 23956768

[B54] Zhang L. Wu J. Ling M. T. Zhao L. Zhao K.-N. (2015). The role of the PI3K/Akt/mTOR signalling pathway in human cancers induced by infection with human papillomaviruses. *Mol. Cancer* 14 87 10.1186/s12943-015-0361-x PMC4498560 26022660

[B55] Zhou C. Y. Yao J. F. Chen X. D. (2002). Expression of matrix metalloproteinase-2, 9 and their inhibitor-TIMP 1,2 in human squamous cell carcinoma of uterine cervix. *Ai Zheng* 21 735–739. 12479097

[B56] Zhou H. Y. Wong A. S. (2006). Activation of p70S6K induces expression of matrix metalloproteinase 9 associated with hepatocyte growth factor-mediated invasion in human ovarian cancer cells. *Endocrinology* 147 2557–2566. 10.1210/en.2005-1404 16469801

[B57] Zigras T. Lennox G. Willows K. Covens A. (2017). Early cervical cancer: current dilemmas of staging and surgery. *Curr. Oncol. Rep.* 19 51 10.1007/s11912-017-0614-5 28664470

