# Supplementary figures and images for: Kaempferia parviflora Extract Exhibits Anti-cancer Activity against HeLa Cervical Cancer Cells
Source: Front Pharmacol. 2017 Sep 11;8:630. doi: 10.3389/fphar.2017.00630 (PMC5600991; doi:10.3389/fphar.2017.00630)

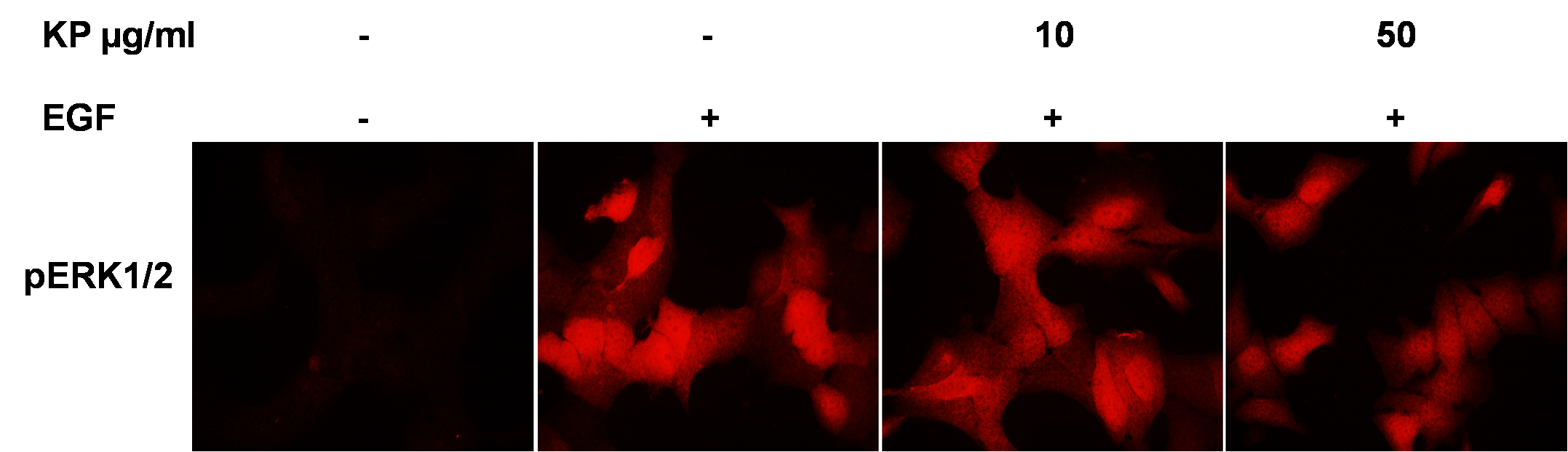

Supplement: Supplementary file 1 [file Image_1.TIF]
